# The transformation of the nuclear nanoarchitecture in human field carcinogenesis

**DOI:** 10.4155/fsoa-2017-0027

**Published:** 2017-05-05

**Authors:** Greta M Bauer, Yolanda Stypula-Cyrus, Hariharan Subramanian, Lusik Cherkezyan, Parvathi Viswanathan, Di Zhang, Radha Iyengar, Saurabh Bagalkar, Justin Derbas, Taylor Graff, Scott Gladstein, Luay M Almassalha, John E Chandler, Hemant K Roy, Vadim Backman

**Affiliations:** 1 Department of Biomedical Engineering, Northwestern University, Evanston, IL 60208, USA; 2 NanoCytomics LLC., Evanston, IL 60201, USA; 3 Section of Gastroenterology, Boston Medical Center/Boston University School of Medicine, Boston, MA 02118, USA; 4 Chemistry of Life Processes Institute, Northwestern University, Evanston, IL 60208, USA

**Keywords:** carcinogenesis, chromatin, field effect, heterogeneity, nanocytology

## Abstract

Morphological alterations of the nuclear texture are a hallmark of carcinogenesis. At later stages of disease, these changes are well characterized and detectable by light microscopy. Evidence suggests that similar albeit nanoscopic alterations develop at the predysplastic stages of carcinogenesis. Using the novel optical technique partial wave spectroscopic microscopy, we identified profound changes in the nanoscale chromatin topology in microscopically normal tissue as a common event in the field carcinogenesis of many cancers. In particular, higher-order chromatin structure at supranucleosomal length scales (20–200 nm) becomes exceedingly heterogeneous, a measure we quantify using the disorder strength (*L_d_*) of the spatial arrangement of chromatin density. Here, we review partial wave spectroscopic nanocytology clinical studies and the technology's promise as an early cancer screening technology.

## Lay abstract

At later stages of cancer development, abnormalities in the nucleus of cancer cells are easily detectable with conventional microscopy techniques. However, there is evidence that nuclear changes are present at a much earlier stage of cancer development. Our lab developed a novel technique, partial wave spectroscopic microscopy, which can identify changes in the nanoscale chromatin topology of otherwise normal looking cells as a common event in the progression to cancer. Here, we provide a review of our technology and its promise as an early cancer screening technology for numerous types of cancer.

Traditional immunohistochemical staining and light microscopy have long been used as a way to identify alterations in nuclear structure during various stages of carcinogenesis. These alterations can have profound effects on many critical cellular functions, such as gene expression and DNA replication. Additionally, the transformation of chromatin structure, including histone modifications and modulation of higher-order chromatin folding, has been associated with abnormal gene regulation in carcinogenesis. Despite this evidence of chromatin level alterations in carcinogenesis, the mechanisms of early nuclear alterations and the accurate identification of such alterations in a clinical setting require further understanding and the development of novel techniques.

Increasing evidence suggests morphological and genetic transformations that are microscopically identifiable also occur during earlier stages in field of carcinogenesis. The field effect (also known as field carcinogenesis, field cancerization or field defect) is the process where the genetic/environmental milieu that produces a neoplastic lesion is present throughout the affected organ. Specifically, these genetic/epigenetic distortions provide a fertile field on which individual tumors eventually arise. Thus, the field effect can be used to identify and study the earliest events in carcinogenesis as some genomic, epigenomic and structural transformations have likely occurred in tissue neighboring the tumor itself [1,2]. The field effect has been observed in colorectal, lung, esophageal, ovarian, cervical, breast, prostate, and head and neck cancers [1]. Field carcinogenesis may explain the recurrence of tumors, whether metachronous or synchronous, and is already practiced in a clinical setting as a diagnostic marker [1,3,4]. In the case of colorectal cancer (CRC), many of the epigenetic [5], proteomic [6] and structural [7,8] alterations have also been reported in the histologically normal appearing colonic mucosa. Therefore, detection of markers representative of field carcinogenesis can be used to screen for and determine the risk of CRC development. However, as these physical transformations occur at the nanoscale, conventional microscopy cannot resolve the early, subtle structural alterations associated with field carcinogenesis.

Consequently, to detect these physical transformations that result from the field effect, we have developed a novel microscopic technique, partial wave spectroscopic (PWS) microscopy, which quantifies the physical properties of cellular structure at the nanoscale (20–200 nm), beyond the resolution limit of conventional microscopy [9]. PWS microscopy measures the spatial heterogeneity of the nanoscale structure by measuring the variations in the refractive index (RI or n) that is quantified through the disorder strength (L_d_). Using PWS microscopy, we have found that an increase in L_d_ is a universal event in many cancers, including colorectal, pancreatic, lung, esophageal, prostate and ovarian cancers [10–16]. Furthermore, this increase in physical heterogeneity develops concurrently with the earliest known genetic events and precedes any known micron-scale alterations detectable by traditional histology [16].

From a biological perspective, the transformation in the cellular structure at these length scales may have a profound impact on a range of cellular processes. For example, in the nucleus, changes in chromatin folding, either through alterations in compaction or the higher-order structure are associated with alterations in transcription [17,18]. While these transformations are often understood and analyzed through their underlying molecular transformation (e.g., differential in histone acetylation and methylation patterns), these molecular transformations converge on changes in the physical structure of chromatin. Classically, these physical transformations have been measured by transmission electron microscopy (TEM). Indeed, TEM has identified nanoscale changes in nuclei at the earliest stages of neoplasia in both the preneoplastic azoxymethane-induced rat model, a commonly used inducible rat model for CRC, as well as in human colonic field carcinogenesis samples [19]. While we found that several organelles display altered ultrastructure, the nucleus in particular is frequently transformed. Image analysis of the nanoscale chromatin distribution (e.g., differential chromatin compaction) was consistent with an increase in disorder strength as measured by PWS microscopy. Other optical techniques, such as quantitative phase microscopy, have observed similar results in field carcinogenesis [20–24]. However, PWS is more sensitive than these methods.

Given their ubiquity and the role of chromatin in critical molecular processes, these results suggest that structural alterations in chromatin represent an early-stage event of carcinogenesis. There are several chromatin-remodeling mechanisms responsible for inducing changes in chromatin structure in early and field carcinogenesis. For example, using qRT-PCR methods, we found the histone deacetylases HDAC1, HDAC2, HDAC3, HDAC5 and HDAC7, all to be upregulated in the field of human CRC [25]. Aberrant regulation of chromatin has been implicated in functional alterations, such as changes in cell cycle, chromosomal stability and gene expression [26]. Recent work, including from our group, has focused on the importance of chromatin structure on transcriptional regulation [27,28]. Furthermore, we found that chromatin heterogeneity, as measured by the disorder strength, correlates with the heterogeneity of global gene expression and the heterogeneity of gene expression within genetic networks. Therefore, PWS microscopy is a powerful tool quantifying nanoscale chromatin structure and subsequent changes in gene expression and their role in the initial stages of carcinogenesis and tumor progression.

In this review, we examine the interconnection of chromatin structure to preneoplastic spectroscopic markers of field carcinogenesis and its concomitant relationship to functional changes in biological processes often implicated in tumorigenesis. We present methodologies to quantify nanoscale nuclear alterations in the preneoplastic cells as well as case studies examining the nuclear disorder strength in human field carcinogenesis. This increase in nuclear disorder strength in carcinogenesis (i.e., topological heterogeneity within chromatin) is a common denominator of multiple molecular pathways, as it is observed in many different and diverse cancer types.

## Results

### PWS microscopy: a tool for measuring the cellular nanoarchitecture

**Figure 1.**
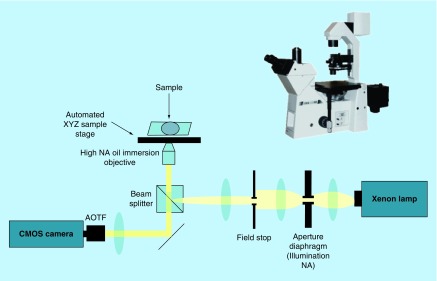
**Partial wave spectroscopic microscope is an epi-illumination bright-field spectroscopic microscope with small illumination numerical aperture, moderate collection and 40× magnification.** Wavelength-resolved image acquisition was performed by using a Xenon white-light lamp illumination followed by spectral filtration of the incident light via an acousto-optical tunable filter. The resulting microscope images (x,y) are obtained at 200 1 nm spaced wavelengths λ of the incident light spanning the spectral range of 500–700 nm, combined into a 3D (x,y,λ) data cube, and used for subsequent spectral analysis. AOTF: Acousto-optical tunable filter; CMOS: Complementary metal-oxide semiconductor; NA: Numerical aperture.

PWS microscopy measurements were performed on in-house designed, high-throughput instrument described in detail in reference (Figure 1) [29]. In brief, the PWS microscope is an epi-illumination bright-field spectroscopic microscope with small illumination numerical aperture NAi = 0.15, moderate collection NAc = 0.6 and 40× magnification (objective lens from LUCPlanFL N, PA, USA). Koehler illumination scheme was implemented for uniformity of incident light intensity throughout the image. To pair spectroscopy with microscopy, wavelength-resolved image acquisition was performed by using a Xenon white-light lamp illumination followed by spectral filtration of the incident light via an acousto-optical tunable filter (HSI-300, Gooch & Housego, FL, USA; filter bandwidth of 3 nm). The resulting microscope images (x,y) are obtained at 200 1 nm spaced wavelengths λ of the incident light spanning the spectral range of 500–700 nm. These images are combined into a 3D (x,y,λ) data cube, which comprised a single PWS measurement used for subsequent spectral analysis.

After the data is collected, spectral noise is removed at each pixel using a sixth order Butterworth filter. Following the spectral de-noising, the L_d_ is calculated at each pixel from the spectra. From these L_d_ images, the average nuclear disorder strength L_d_ is calculated by segmentation for a particular patient sample. The statistical significance of difference between L_d_ values measured from patients falling into two different diagnostic categories was evaluated through the calculated effect size (the difference between the means of two groups divided by the cumulative SD) and the p-value.

The resolution of conventional microscopic imaging technique has a fundamental diffraction limit of approximately 200 nm. In order to assess nanoscale topology, PWS microscopy couples spectroscopy with microscopy. Since RI is proportional to the local density (ρ) of macromolecules (proteins, lipids, DNA and RNA): n = n_water_ + αρ with α denoting specific refraction increment of 0.18 ml/g [30], the spatial organization of macromolecular density can be measured through spatial variations of cellular RI.

We have previously demonstrated that a spectroscopic microscope, configured to detect interference spectra of backscattered light in the far zone, can quantify the statistics of nanometer scale RI distribution via the spectral variance (Σ^2^) of the acquired bright-field image [31]. Further, we have determined that Σ^2^ can sense RI fluctuations at any spatial frequency whatsoever and its length-scale sensitivity range is limited only by the S/N of the instrument [32]. In addition to its unique sensitivity to subdiffractional length scales, Σ^2^ is not sensitive to structural alterations at large length scales [31,32], making it an ideal marker to measure the subdiffractional intracellular ultrastructure within cells.

In brief, for each pixel (x,y) within the microscope image, an interference signal R(λ) between a reference wave and scattering from all of the RI variations (macromolecular density distributions) within a cylindrical volume defined by the spatial coherence in the transverse plane and the cell thickness longitudinally is recorded. Owing to the geometry of the sample on the glass slide, the cellular RI is matched at one surface and mismatched at the other. The Σ^2^ of the recorded spectrum is sensitive to the organization and distribution of macromolecular structures within the length scales that range between 20 and 200 nm [32].

For small correlation lengths (l_C_), the SD of the interference spectrum (∑) is proportional to σ_p_ * l_c_ * L, where σ_p_ is the SD of mass density fluctuations within the cell and L is the cell thickness. For each resolution pixel, Σ is converted into the disorder strength of the macromolecular density distribution by normalizing the overall thickness of the cells at each pixel, L_d_(x, y) ∝ σ_p_ * l_c_

Thus, the disorder strength L_d_(x, y) is the product of the variations in macromolecular compaction (assessed via σ_p_) and their characteristic size (assessed via l_C_). The integration of these two transformations is a cumulative measure of macromolecular folding [33]. Therefore, in the context of the cellular nucleus, L_d_ measures the variations in the nanoscale compaction of chromatin [33].

### Nanoarchitectural transformation in early carcinogenesis

**Figure 2.**
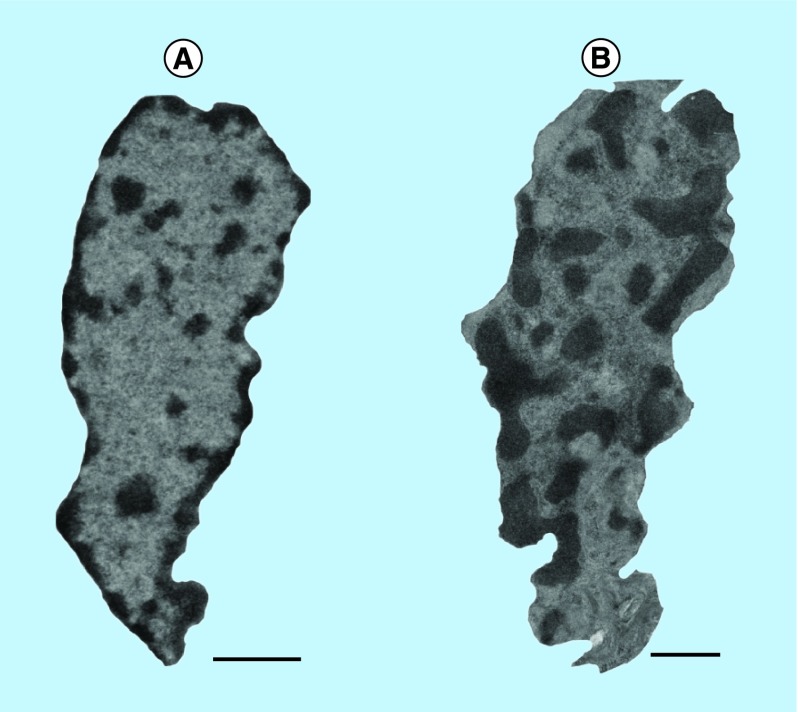
Pre-neoplastic cells have significant nanoscale physical alterations in the nucleus. Transmission electron microscopy micrographs of rectal cell nuclei of **(A)** a healthy patient and a **(B)** patient harboring tumor elsewhere in colon, representing the field effect of carcinogenesis. Scale bar corresponds to 1 μm.

Electron microscopy has long been the gold standard for detecting nanoscale changes in cellular structure. To first measure nanoscale changes in the nucleus during early carcinogenesis, TEM image analysis was performed on cells using colorectal field carcinogenesis as a model in humans. In this analysis, we found that there are significant physical alterations in the chromatin of preneoplastic cells that would otherwise be indistinguishable by standard histology and conventional light microscopy (Figure 2). While these ultrastructures can be identified by TEM, there are significant downsides to using electron microscopy as a diagnostic or prediagnostic screening tool in the clinical setting. Primarily, TEM imaging has high costs per sample and has significant restrictions in availability due to the difficult and lengthy sample preparation required. Furthermore, the inherent complexity of TEM preparation necessitates expert use and analysis, which consequently prevents the technique from being used as a high-throughput diagnostic tool. Optical microscopy techniques are a much more practical choice for clinical diagnostics due to their relatively low cost, high throughput and ease of use. As discussed above, however, traditional light microscopy is diffraction limited and thus cannot provide information on the organization of structures below 200 nm. PWS microscopy addresses this problem by providing a nanoscale-sensitive technique as an efficient, low cost and nanoscale-sensitive cancer screening assay.

**Figure 3.**
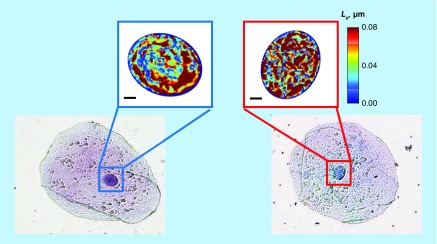
**Representative transmission bright-field microscope images (bottom row) of histologically normal buccal cells from a healthy patient (left) and a patient with lung cancer (right).** Nuclei were selected using the transmission images after which their disorder strength distribution was obtained (top row). Scale bar corresponds to 2 μm.

Using PWS microscopy, we have shown that, similar to commonly identified histological biomarkers, nuclear nanoscale alterations are a common denominator at the earliest stages in numerous types of solid tumors (Figure 3). In particular, we have previously shown using PWS microscopy that L_d_ is a measure of early neoplastic changes in cells, which quantifies the spatial heterogeneity of mass density at subdiffractional length scales (∼20–200 nm). Table 1 summarizes the published L_d_ results from over 1300 human patient samples across seven different types of cancers. As seen, PWS microscopy measurements on clinical samples have shown an increase in L_d_ to be a universal event in multiple cancers, including colorectal, pancreatic, thyroid, lung, esophageal, prostate and ovarian cancers [10–16,34,35]. As an optical microscopy technique, PWS microscopy easily allows the user to localize L_d_ measurements within different cellular compartments of interest, giving quantitative values of nanoscale structural changes directly related to different organelles and in relation to various molecular functions [36]. For example, squamous epithelial cells deposited onto glass slides are thin, flat and have a large cytoplasmic area. Nevertheless, since PWS microscopy obtains spatially resolved maps of L_d_, the disorder strength of the nucleus can be calculated independent of the ultrastructural transformation that occurs in neighboring organelles or within the cytoskeleton. Additionally, nuclear segmentation algorithms can be developed to automatically detect nuclear L_d_, even with the context of the structural transformation occurring in the whole cell. [37].

**Table 1.** **Summary of partial wave spectroscopic microscopy measurements in human field carcinogenesis.**

**Tumor**	**Surrogate site**	**Effect size in nucleus (%)**	**p-value**	**Number of patients**	**Ref.**
Colon	Rectum	144	<0.01	343	[11,14,16]
Ovary	Endocervix	108	<0.01	30	[10]
Thyroid	Histo-normal thyroid	85	<0.05	18	[35]
Pancreas	Duodenum	109	<0.01	35	[16]
Distal esophagus	Proximal esophagus	54	= 0.11	26	[12]
Prostate	Progressors versus nonprogressors	116	<0.05	58	[13]
Lung	Oral mucosa	104	<0.001	825	[15,34]

An increase in structural heterogeneity was observed in nonmalignant cells obtained from cancer patients. In a cohort totaling over 700 patient samples obtained from seven different organs, topological heterogeneity increased in patients with malignancy. Within these studies, analysis of the nuclear structure was performed on 252 patients showing an increase in chromatin physical heterogeneity as measured by L_d_ across multiple cancer subtypes.

**Figure 4.**
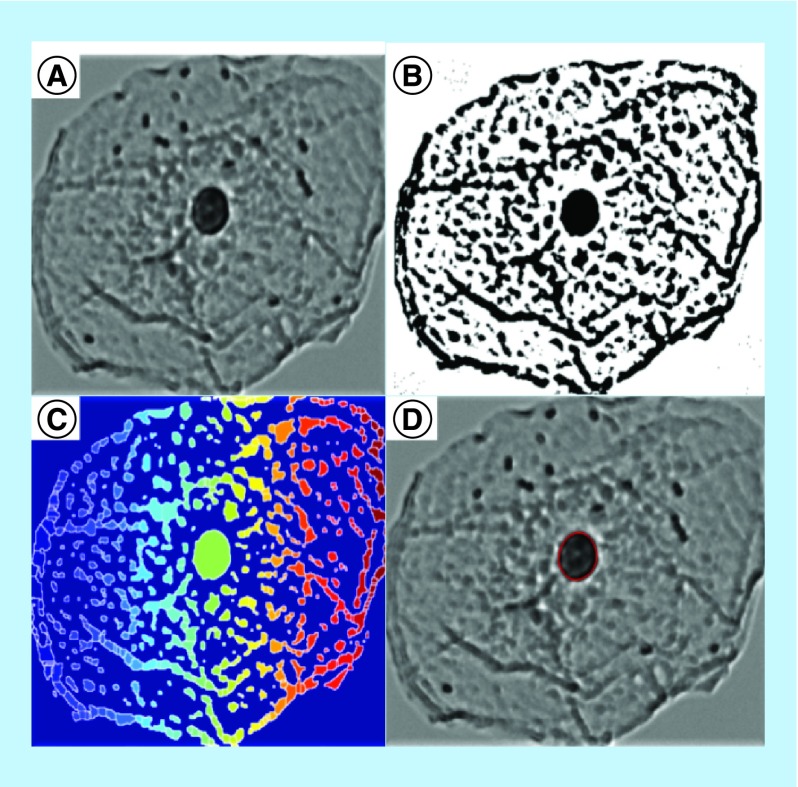
**Nuclei segmentation of an isolated, representative buccal cell.** **(A)** Isolated buccal cell imaged with light transmission, **(B)** maximum entropy thresholding, **(C)** watershed segmentation and **(D)** with resulting segmented nuclei outlined.

**Figure 5.**
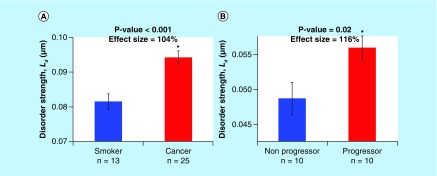
**Nuclear L_d_ increases in human field carcinogenesis.** **(A)** The nuclear L_d_ was calculated from buccal cells obtained from the oral mucosa of smokers (control) compared with patients harboring lung cancer. Nuclear L_d_ is increased significantly in buccal cells from cancer patients (effect size: 1.04, p-value < 0.001, n = 38 patients total). Panel **(B)** shows the nuclear L_d_ calculated form FFPE prostrate tissue samples from progressors and nonprogressors. FFPE slides were stained with low concentration of H&E to provide contrast. Nuclear L_d_ is significantly increased in the patients who went on to develop the disease (progressors), compared with the benign form (nonprogressors). Therefore, nuclear L_d_ can be a useful marker in lung cancer screening, while the prostate nuclear L_d_ data represent the first test to differentiate between those who will actually go onto present malignant forms of the disease, which will correspond to alterations in biological pathways (i.e., chromatin structure and gene expression). Error bars represent standard error. FFPE: Formalin-fixed and paraffin embedded; H&E: Hematoxylin and eosin.

In the majority of cancer types shown in Table 1 (colon, pancreas, thyroid and ovary), the PWS measurements were performed on tissue monolayers that were primarily comprised of columnar cells (colon – rectal cells, pancreas – duodenal cells, thyroid – thyroid cells and ovary – endocervical cells) in which the nucleus occupied >80% of the cytology [38]. Hence, the effect size and p-values reported in Table 1 are principally the changes in nuclear L_d_ in these organ types. However, in the case of other cancer types (lung and esophagus), the PWS measurements were obtained from isolated squamous cells (lung – buccal cells and esophagus – proximal esophageal cells). In these cell types, the nucleus in the cells we measured occupied a much smaller portion of the total cell. For example, the nuclei in the buccal cells occupied less than 25% of the total cell volume. Thus, to better understand the changes in nuclear L_d_ in squamous cells, we performed an independent study on 38 smokers with (25 subjects) and without (13 subjects) lung cancer. In this study, an image processing algorithm was used to automatically calculate the nuclear L_d_ by measuring the changes to the nuclear nanostructure of oral epithelial (buccal) cells (Figure 4). To examine the differences in buccal nuclear L_d_ due to lung cancer, we compared the nuclei from microscopically normal-appearing buccal cells obtained from smokers with lung cancer to those of smokers who were neoplasia-free as confirmed by upper endoscopy. There was a significant increase in the nuclear L_d_ in buccal cells obtained from patients with cancer compared with those from control patients (effect size: 104%; p-value < 0.001) (Figure 5A). Importantly, the nuclear L_d_ and the cellular L_d_ (calculated as the average L_d_ from the nuclear and perinuclear area of the cell) had a very strong correlation (R > 80%). This increase in nuclear L_d_ is similar to the previously reported studies on human buccal cells in over 825 patients (including nonsmokers, smokers with and without lung cancer etc.) [15,34]. These results validate that PWS microscopy was able to detect changes to the nuclear nanostructure that provided indication of an early disease stage not detectable by traditional histological methods distal to the site of the tumor.

In addition to its ability to identify patients with early disease in multiple cancer types, we have also shown the utility of PWS microscopy as a prognostic tool to measure disease aggressiveness [13]. As an example of this approach, we utilized PWS microscopy to measure topological alterations in the nucleus of progressor prostate cancer patients in comparison to nonprogressor controls. Specifically, we studied the relationship between chromatin structure and prostate cancer aggressiveness by performing PWS microscopy measurements on prostate tissue sections obtained from patients by biopsy. Significantly, we observed an increase in L_d_ within the nucleus of future progressors compared with that measured in nonprogressors (effect size: 116%; p-value < 0.05; n = 20 patients) (Figure 5B) [13]. In this analysis, we examined nuclear L_d_ differences in these tissue sections obtained from the histologically normal prostate epithelium. Formalin-fixed and paraffin embedded slides were stained with low concentration of hematoxylin and then segmented using the automated nuclear segmentation algorithm to automatically analyze the nucleus. Given the sensitivity to the resulting prostate cancer progression, PWS microscopy could be an important tool in prostate cancer screening, as the current prostate-specific antigen test cannot differentiate between men who will go on to develop aggressive cancer.

## Discussion

In summary, these studies indicate that the nanoscopic transformation of the nucleus is a universal event in early carcinogenesis seen across many cancer types including colon, lung, pancreatic, esophageal, thyroid and ovarian cancers [11–16,34,35,38] and is potentially linked to cancer cell aggressiveness [13]. This convergence of the physical structure of chromatin with the molecular transformations frequently observed in carcinogenesis (e.g., aberrant transcriptional alterations, epigenetic changes in histones, etc.) suggests that the topology of chromatin may be more than a marker of the molecular transformation of cells during malignancy. Indeed, this evidence suggests that deformation of chromatin folding could play a role in driving the observed molecular transformations. Owing to the sensitivity of PWS microscopy to measure these physical alterations within the nucleus, this technology can provide a noninvasive method for early cancer risk stratification; provide a means to obtain prognostic information on tumor aggressiveness; and shed light on the interplay between physical structure and molecular transformation in oncogenesis. Given the importance of chromatin structure on biological processes and the observation of its transformation during cancer initiation and progression, we utilized PWS microscopy to examine the transformation of nuclear nanostructure as measured by our optical measurements during human field carcinogenesis. Abnormalities in nuclear organization are one of the most definitive markers of dysplasia and malignancy and have been universally used by pathologists for decades. Of note, microscopic nuclear alterations, such as nuclear atypia and abnormal nuclear texture (microscale chromatin heterogeneity), are consistently observed by pathologists in the absolute majority of solid tumors and are convergent physical phenomena in multiple molecular carcinogenesis pathways. We have reported that similar alterations in the nanoscopic heterogeneity of chromatin structure develop at a predysplastic stage of carcinogenesis (e.g., field carcinogenesis) in microscopically normal-appearing cells. For example, in colorectal tissue monolayers measured from colonic crypts, the nucleus occupied a significant portion of the columnar cells (>80%) forming the colonic crypts. Thus, changes in nuclear L_d_ during carcinogenesis would dominate the measured PWS signal. Furthermore, as PWS measures fluctuations in RI, it would be expected that dense regions of nucleic acids, ie., chromatin, would dominate the PWS signal. Indeed, a cell line study showed that while the cytoskeleton also contributes to increased L_d_, the nuclear signal dominates, and nuclear differences in heterogeneity are the driver of tumorigenicity between different HT-29 cell types [39,40].

Changes detected by PWS microscopy are inherently nanoscopic (on the order of 20–200 nm) and have been independently identified on electron microscopy. [19,41–44]. Within chromatin, the nanoscopic length scales to which PWS microscopy is sensitive are those that have been identified to be critical regulators of gene transcription. In particular, these lengths span from supranucleosomal organization all the way into higher-order looping domains. Optical techniques such as PWS microscopy that can quantify the alterations in chromatin folding at this nanoscale range can serve as sensitive, cost-efficient approaches for cancer screening. Likewise, given its ubiquity in early carcinogenesis, this physical transformation may in fact serve as a universal marker for carcinogenesis (it has been invariably observed to increase in >700 patients across seven different types of cancers (Table 1; [10–16]). Thus, this increase in nuclear nanoscale structural heterogeneity, as measured by PWS microscopy, could be the common denominator of multiple unique molecular pathways associated with distinct cancer types and patients. In order to specifically measure the transformation of the chromatin nanostructure in early carcinogenesis, we analyze topological changes within the nucleus of cells for colon, ovarian, thyroid, pancreatic, esophageal, prostate and lung cancers. Given the near-universal observation of this transformation, the disorder strength can be used as a universal biomarker for preneoplastic changes. These results indicate not only the utility of optical biomarkers for cancer screening, but also the underlying drivers of cancerous processes, such as chromatin alterations.

Alterations in chromatin compartmentalization and folding can influence gene expression in several ways, such as by nonlinearly changing the accessibility of chromatin binding sites to transcription factors or by modulating the diffusivity and binding affinity of enzymes such as RNA polymerases. Therefore, there is a significant effort to understand how the differential packaging of the genome impacts cellular processes as well as how these functions are altered during cancer [45,46]. The differential compaction of chromatin is influenced by a wide array of factors that range from molecular transformations (e.g., histone deacetylase activity) to the physio-chemical (e.g., local osmolarity, pH and ionic conditions). Classically, epigenetics has been studied through analysis of localized histone or DNA chemical modifications. While the role of these molecular transformations is indisputable, the overall folding of the genome likely depends on the confluence of numerous regulators, some of which cannot be identified by *ex vivo* molecular techniques. For example, deacetylation of histones creates a positive charge on the histones, increasing affinity for the negatively charged DNA. However, this modification in affinity can potentially be achieved by altering the counter-ion concentrations surrounding chromatin (K^+^ or Ca^2+^) [36]. While there are molecular methods to directly analyze and measure the alterations in histone acetylation or other chromatin-modifying enzymes in carcinogenesis, there are a number of challenges to directly monitor these additional physio-chemical regulators of chromatin. As a result, indirect measurements of the overall nanoscopic folding of chromatin, such as those provided by TEM or PWS microscopy, could shed light on the convergence of these molecular and physio-chemical forces during early carcinogenesis [25,47].

In this context, it may be important to consider the physical folding of chromatin in and of itself as a regulator of gene expression and a potential contributor to oncogenesis [48]. This may allow us to better understand the overall integration between the heterogeneous epigenetic transformations that occur during oncogenesis. In this view, changes in DNA methylation (CpG islands), histone modifications, and mutations in both higher-order chromatin modulators (cohesins, condensins) and histone modifying enzymes (HDACs, SWItch/Sucrose nonfermentable enzymes) could be convergent on global changes that act at the level of a genomic ‘folding code’ [49–53]. Interestingly, PWS microscopy has shown that some of these alterations are manifested in transformation of the physical structure of chromatin [25,54,55].

Furthermore, the modulation of this higher order folding code can be viewed in the context of increasing interest in targeting epigenetic mechanisms for cancer therapeutics. Unlike genetic modifications that largely cannot be reversed, epigenetic therapies present the opportunity for reversible regulation of gene expression by altering the epigenetic state of cells. To date, clinical trials have found some success at this epigenetic level through the use of DNA demethylating agents, HDAC inhibitors and the histone methyltransferase EZH2 [56–61]. Indeed, the success of these interventions is due to the role that epigenetic mutations and modifications have in conferring resistance to traditional chemotherapeutics. At a broader level, this epigenetically driven resistance may be in part due to global alterations in chromatin topology producing increased access to the genomic information space. Thus, epigenetic therapies that regulate both local modifications to critical genes and the overall folding of the genome could have a considerable effect on delaying the emergence of chemoresistance.

## Future perspective

In summary, in this review we demonstrate the near-universal link between nanoscopic changes in chromatin physical topology in early tumorigenesis in colorectal, lung, esophageal, ovarian, cervical, breast, prostate, and head and neck cancers. These previously unidentified nanoscopic transformations mirror the widely recognized microscopic alterations recognized both in dysplastic and malignant cells. This observation of increased heterogeneity of higher-order chromatin structure at supranucleosomal length scales, as measured by PWS microscopy, is a common denominator of multiple molecular carcinogenesis pathways and may serve as a marker of early carcinogenesis across multiple cancer types and as a prognostic indicator of aggressiveness in prostate cancer. Given that these alterations in higher-order chromatin structure could alter molecular function, they could also potentially be used to measure chemoevasion. If this can be demonstrated, PWS microscopy would have significant clinical relevance to tailoring epigenetic therapies and personalized medicine as it would provide a means to assess premalignant risk, measure tumor aggressiveness and assess chemoevasive potential. Furthermore, our group has recently developed a live cell PWS system [55], which can address the question of how nuclear nanostructure is organized in live cells, unobscured by potential artifacts of fixation and the real-time functional consequences in human-derived cell lines. While fixed cell PWS nanoscopy is expected to be vital for cost-effective cancer screening and risk stratification, this live cell extension of PWS microscopy can have significant implications and applications in the field of personalized medicine and tailoring cancer therapeutics to an individual's tumor cells.

**Executive summary**

**There is a need for the earlier detection of morphological alterations associated with cancer progression**

- Morphological and genetic transformations also occur during early stages in the field of carcinogenesis before they are microscopically identifiable.
- The field effect can be used to identify and study these earliest events in carcinogenesis.
- Detection of markers representative of field carcinogenesis can be used to screen for and determine the risk of cancer development. However, as these physical transformations occur at the nanoscale, conventional microscopy cannot resolve these structural alterations.

**Partial wave spectroscopic microscopy can uniquely detect the nanoscale physical transformations associated with the field effect**

- Partial wave spectroscopy (PWS) quantifies the physical properties of cellular structure at the nanoscale (20–200 nm), beyond the resolution limit of conventional microscopy.
- Using PWS microscopy, we have found that an increase in L_d_ (spatial heterogeneity of the nanoscale structure) is a universal event in many cancers, including colorectal, pancreatic, lung, esophageal, prostate and ovarian cancers.
- An increase in physical heterogeneity develops at the same time as the earliest known genetic events and precedes any known alterations detectable by conventional histology.
- Image analysis of the nanoscale chromatin distribution was consistent with an increase in L_d_ as measured by PWS microscopy.

**PWS microscopy measures disease aggressiveness**

- PWS microscopy was applied to measure topological alterations in the nucleus of progressor prostate cancer patients in comparison to nonprogressor controls.
- We observed an increase in L_d_ within the nucleus of future progressors compared with that measured in nonprogressors.
- Unlike the current prostate-specific antigen test, PWS microscopy was able to differentiate between those patients who will go on to develop aggressive cancer and those who will not.

**Conclusion**

- PWS microscopy provides a nanoscale-sensitive technique as an efficient, low cost and nanoscale-sensitive cancer screening assay.
- The increase in nuclear nanoscale structural heterogeneity analyzed by PWS microscopy may be the common denominator of multiple unique molecular pathways associated with distinct cancer types.
- PWS microscopy can offer a noninvasive method for early cancer risk stratification, obtain prognostic information on tumor aggressiveness and examine the relationship between physical structure and molecular transformation associated with carcinogenesis.
- These alterations in higher-order chromatin structure could also potentially be used to measure chemoevasion. Thus, PWS microscopy would be extremely relevant in furthering the field of personalized medicine as it could assess premalignant risk, tumor aggressiveness and chemoevasive potential.

## References

[1] Braakhuis BJ, Tabor MP, Kummer JA, Leemans CR, Brakenhoff RH (2003). A genetic explanation of Slaughter's concept of field cancerization: evidence and clinical implications. *Cancer Res.*.

[2] Dakubo GD, Jakupciak JP, Birch-Machin MA, Parr RL (2007). Clinical implications and utility of field cancerization. *Cancer Cell Int.*.

[3] Backman V, Roy HK (2011). Light-scattering technologies for field carcinogenesis detection: a modality for endoscopic prescreening. *Gastroenterology*.

[4] Foo J, Leder K, Ryser MD (2014). Multifocality and recurrence risk: a quantitative model of field cancerization. *J. Theor. Biol.*.

[5] Paun BC, Kukuruga D, Jin Z (2010). Relation between normal rectal methylation, smoking status, and the presence or absence of colorectal adenomas. *Cancer*.

[6] Polley AC, Mulholland F, Pin C (2006). Proteomic analysis reveals field-wide changes in protein expression in the morphologically normal mucosa of patients with colorectal neoplasia. *Cancer Res.*.

[7] Radosevich AJ, Rogers JD, Turzhitsky V (2012). Polarized enhanced backscattering spectroscopy for characterization of biological tissues at subdiffusion length-scales. *IEEE J. Sel. Top. Quantum Electron.*.

[8] Roy HK, Turzhitsky V, Kim Y (2009). Association between rectal optical signatures and colonic neoplasia: potential applications for screening. *Cancer Res.*.

[9] Subramanian H, Pradhan P, Liu Y (2008). Optical methodology for detecting histologically unapparent nanoscale consequences of genetic alterations in biological cells. *Proc. Natl Acad. Sci. USA*.

[10] Damania D, Roy HK, Kunte D (2013). Insights into the field carcinogenesis of ovarian cancer based on the nanocytology of endocervical and endometrial epithelial cells. *Int. J. Cancer*.

[11] Damania D, Roy HK, Subramanian H (2012). Nanocytology of rectal colonocytes to assess risk of colon cancer based on field cancerization. *Cancer Res.*.

[12] Konda VJ, Cherkezyan L, Subramanian H (2013). Nanoscale markers of esophageal field carcinogenesis: potential implications for esophageal cancer screening. *Endoscopy*.

[13] Roy HK, Brendler CB, Subramanian H (2015). Nanocytological field carcinogenesis detection to mitigate overdiagnosis of prostate cancer: a proof of concept study. *PLoS ONE*.

[14] Roy HK, Damania DP, Delacruz M (2013). Nano-architectural alterations in mucus layer fecal colonocytes in field carcinogenesis: potential for screening. *Cancer Prev. Res. (Phila.)*.

[15] Roy HK, Subramanian H, Damania D (2010). Optical detection of buccal epithelial nanoarchitectural alterations in patients harboring lung cancer: implications for screening. *Cancer Res.*.

[16] Subramanian H, Roy HK, Pradhan P (2009). Nanoscale cellular changes in field carcinogenesis detected by partial wave spectroscopy. *Cancer Res.*.

[17] Li G, Reinberg D (2011). Chromatin higher-order structures and gene regulation. *Curr. Opin. Genet. Dev.*.

[18] Hayashi MT, Masukata H (2011). Regulation of DNA replication by chromatin structures: accessibility and recruitment. *Chromosoma*.

[19] Cherkezyan L, Stypula-Cyrus Y, Subramanian H (2014). Nanoscale changes in chromatin organization represent the initial steps of tumorigenesis: a transmission electron microscopy study. *BMC Cancer*.

[20] Bista RK, Wang P, Bhargava R (2012). Nuclear nano-morphology markers of histologically normal cells detect the “field effect” of breast cancer. *Breast Cancer Res. Treat.*.

[21] Hartman DJ, Krasinskas AM, Uttam S (2014). Assessment of nuclear nanomorphology marker to improve the detection of malignancy from bile duct biopsy specimens. *Am. J. Clin. Pathol.*.

[22] Robles FE, Zhu Y, Lee J, Sharma S, Wax A (2010). Detection of early colorectal cancer development in the azoxymethane rat carcinogenesis model with Fourier domain low coherence interferometry. *Biomed. Opt. Express.*.

[23] Sridharan S, Macias V, Tangella K, Kajdacsy-Balla A, Popescu G (2015). Prediction of prostate cancer recurrence using quantitative phase imaging. *Sci. Rep.*.

[24] Uttam S, Pham HV, Laface J (2015). Early prediction of cancer progression by depth-resolved nanoscale mapping of nuclear architecture from unstained tissue specimens. *Cancer Res.*.

[25] Stypula-Cyrus Y, Damania D, Kunte DP (2013). HDAC up-regulation in early colon field carcinogenesis is involved in cell tumorigenicity through regulation of chromatin structure. *PLoS ONE*.

[26] Kagami Y, Yoshida K (2016). The functional role for condensin in the regulation of chromosomal organization during the cell cycle. *Cell. Mol. Life Sci.*.

[27] Matsuda H, Putzel GG, Backman V, Szleifer I (2014). Macromolecular crowding as a regulator of gene transcription. *Biophys. J.*.

[28] Almassalha LM, Tiwari A, Ruhoff PT (2017). The global relationship between chromatin physical topology, fractal structure, and gene expression. *Sci. Rep.*.

[29] Chandler JE, Subramanian H, Maneval CD, White CA, Levenson RM, Backman V (2013). High-speed spectral nanocytology for early cancer screening. *J. Biomed. Opt.*.

[30] Davies HG, Wilkins MHF, Chayen J, La Cour LF (1954). The use of interference microscope to determine dry mass in living cells and as a quantitative cytochemical method. *Q. J. Microsc. Sci.*.

[31] Cherkezyan L, Capoglu I, Subramanian H (2013). Interferometric spectroscopy of scattered light can quantify the statistics of subdiffractional refractive-index fluctuations. *Phys. Rev. Lett.*.

[32] Cherkezyan L, Subramanian H, Backman V (2014). What structural length scales can be detected by the spectral variance of a microscope image?. *Opt. Lett.*.

[33] Kim JS, Pradhan P, Backman V, Szleifer I (2011). The influence of chromosome density variations on the increase in nuclear disorder strength in carcinogenesis. *Phys. Biol.*.

[34] Subramanian H, Viswanathan P, Cherkezyan L (2016). Procedures for risk-stratification of lung cancer using buccal nanocytology. *Biomed. Opt. Express.*.

[35] Damania D (2013). Understanding alterations in cell nano-architecture during early carcinogenesis using optical microscopy. http://adsabs.harvard.edu/abs/2013PhDT........60D.

[36] Chandler JE, Stypula-Cyrus Y, Almassalha L (2016). Colocalization of cellular nanostructure using confocal fluorescence and partial wave spectroscopy. *J. Biophotonics*.

[37] Miao Q, Derbas J, Eid A, Subramanian H, Backman V (2016). Automated cell selection using support vector machine for application to spectral nanocytology. *BioMed Res. Int.*.

[38] Damania D, Roy HK, Kunte D (2013). Insights into the field carcinogenesis of ovarian cancer based on the nanocytology of endocervical and endometrial epithelial cells. *Int. J. Cancer*.

[39] Damania D, Subramanian H, Tiwari AK (2010). Role of cytoskeleton in controlling the disorder strength of cellular nanoscale architecture. *Biophys. J.*.

[40] Michor F, Liphardt J, Ferrari M, Widom J (2011). What does physics have to do with cancer?. *Nat. Rev. Cancer*.

[41] Mehta A, Dobersch S, Romero-Olmedo AJ, Barreto G (2015). Epigenetics in lung cancer diagnosis and therapy. *Cancer Metastasis Rev.*.

[42] Rauscher GH, Kresovich JK, Poulin M (2015). Exploring DNA methylation changes in promoter, intragenic, and intergenic regions as early and late events in breast cancer formation. *BMC Cancer*.

[43] Wu J, Keng VW, Patmore DM (2016). Insertional mutagenesis identifies a STAT3/Arid1b/beta-catenin pathway driving neurofibroma initiation. *Cell Rep.*.

[44] You JS, Jones PA (2012). Cancer genetics and epigenetics: two sides of the same coin?. *Cancer Cell*.

[45] Gibcus JH, Dekker J (2013). The hierarchy of the 3D genome. *Mol. Cell*.

[46] Lieberman-Aiden E, Van Berkum NL, Williams L (2009). Comprehensive mapping of long-range interactions reveals folding principles of the human genome. *Science*.

[47] Minucci S, Pelicci PG (2006). Histone deacetylase inhibitors and the promise of epigenetic (and more) treatments for cancer. *Nat. Rev. Cancer*.

[48] Almassalha LM, Bauer GM, Chandler JE (2016). The greater genomic landscape: the heterogeneous evolution of cancer. *Cancer Res.*.

[49] Jones PA, Baylin SB (2007). The epigenomics of cancer. *Cell*.

[50] Mazor T, Pankov A, Song JS, Costello JF (2016). Intratumoral heterogeneity of the epigenome. *Cancer Cell*.

[51] Hill VK, Kim JS, Waldman T (2016). Cohesin mutations in human cancer. *Biochim. Biophys. Acta*.

[52] Johnstone RW (2002). Histone-deacetylase inhibitors: novel drugs for the treatment of cancer. *Nat. Rev. Drug Discov.*.

[53] Marquez-Vilendrer SB, Thompson K, Lu L, Reisman D (2016). Mechanism of BRG1 silencing in primary cancers. *Oncotarget*.

[54] Wali RK, Momi N, Dela Cruz M (2016). Higher-order chromatin modulator cohesin SA1 is an early biomarker for colon carcinogenesis: race-specific implications. *Cancer Prev. Res. (Phila.)*.

[55] Almassalha LM, Bauer GM, Chandler JE (2016). Label-free imaging of the native, living cellular nanoarchitecture using partial-wave spectroscopic microscopy. *Proc. Natl Acad. Sci. USA*.

[56] Brown R, Curry E, Magnani L, Wilhelm-Benartzi CS, Borley J (2014). Poised epigenetic states and acquired drug resistance in cancer. *Nat. Rev. Cancer*.

[57] Nebbioso A, Carafa V, Benedetti R, Altucci L (2012). Trials with ‘epigenetic’ drugs: an update. *Mol. Oncol.*.

[58] Nervi C, De Marinis E, Codacci-Pisanelli G (2015). Epigenetic treatment of solid tumours: a review of clinical trials. *Clin. Epigenetics*.

[59] West AC, Johnstone RW (2014). New and emerging HDAC inhibitors for cancer treatment. *J. Clin. Invest.*.

[60] Mccabe MT, Creasy CL (2014). EZH2 as a potential target in cancer therapy. *Epigenomics*.

[61] Mccabe MT, Ott HM, Ganji G (2012). EZH2 inhibition as a therapeutic strategy for lymphoma with EZH2-activating mutations. *Nature*.

